# Chronic Lymphocytic Leukemia in Neurofibromatosis Type 1 Patients: Case Report and Literature Review of a Rare Occurrence

**DOI:** 10.7759/cureus.14258

**Published:** 2021-04-02

**Authors:** Philip R Cohen

**Affiliations:** 1 Dermatology, San Diego Family Dermatology, National City, USA

**Keywords:** glioma, iris, leukemia, lisch, lymphocytic, neurofibromatosis, axillary, café-au-lait macule, chronic, freckling

## Abstract

Neurofibromatosis type 1 (NF1) is an autosomal dominant genodermatosis that may also occur as the result of a spontaneous mutation. The diagnosis can be established by the presence of two of the seven National Institutes of Health (NIH) diagnostic criteria; several dermatologic manifestations are NIH criteria used to establish the diagnosis: axillary and inguinal freckling, café-au-lait macules, and neurofibromas. Mucosal evaluation of the eyes may detect a fourth criteria: pigmented iris hamartomas (Lisch nodules). The remaining NIH criteria include optic path glioma, distinctive osseus lesions, and a positive family history of the condition. A breast cancer 2 (BRCA2) positive woman with NF1 and chronic lymphocytic leukemia is described. Patients with NF1 have an increased lifetime risk to develop breast cancer, gastrointestinal stromal tumor, malignant glioma, malignant peripheral nerve sheath tumor, and rhabdomyosarcoma. Chronic lymphocytic leukemia occurring in NF1 patients is rare; including my female patient reported in this paper, chronic lymphocytic leukemia has only been reported in three individuals with NF1--two women and one man. The man and the other woman presented with advanced chronic lymphocytic leukemia and treatment with antineoplastic therapy at diagnosis; the man achieved clinical remission and the woman passed away from complications associated with therapy-refractory progression of her leukemia. My female patient required treatment 41 months after diagnosis and had a good clinical response; she has been without significant disease progression for 34 months. Similar to NF1, breast cancer 1 (BRCA1) and BRCA2 mutations are associated with an increased lifetime risk of developing cancer--particularly breast and ovarian carcinoma. An increased risk of chronic lymphocytic leukemia has also been demonstrated in patients with mutations of either BRCA1 or BRCA2. Also, albeit uncommon, either BRCA1 or BRCA2 mutation has been detected in women with NF1 who develop breast cancer. In conclusion, the development of chronic lymphocytic leukemia in NF1 patients may be coincidental and not associated with the underlying genodermatosis; however, the occurrence of chronic lymphocytic leukemia in my patient with NF1, in part, may be related to her BRCA2 positivity.

## Introduction

Neurofibromatosis type 1 (NF1), previously referred to as von Recklinghausen disease, is an autosomal dominant genodermatosis that has been observed to occur in approximately one of every 2000 to 6000 births; however, spontaneous mutations account for about 50% of affected individuals. The condition results from a mutation in the NF1 gene which is located on the long arm of chromosome 17 (17q11.2); the NF1 gene codes for neurofibromin, a tumor suppressor protein that regulates cell growth and tumorigenesis. In addition to cutaneous features, NF1 can have multiple other potential manifestations: cardiovascular, endocrinologic, neurologic, oncologic, ophthalmologic, and orthopedic [1-3].

Chronic lymphocytic leukemia is a lymphoid malignancy that typically occurs in older individuals. It is characterized by the clonal expansion of mature cluster of differentiation five (CD5) positive cells in the blood (with more than 5000 lymphocytes per cubic millimeter), bone marrow, and lymphoid tissue. There have been substantial advances in the treatment of chronic lymphocytic leukemia patients; in contrast to older chronic lymphocytic leukemia therapies such as chemotherapy and antibodies to cluster of differentiation 20 (CD20), newer agents are able to target the molecular pathogenesis of chronic lymphocytic leukemia: Bruton’s tyrosine kinase (BTK) inhibitors (such as ibrutinib, acalabrutinib, and zanubrutinib), isoform-selective phosphatidylijnositol 3-kinase (PI3K) inhibitors (such as idelalisib and duvelirib), and B cell lymphoma 2 (BCL2) antagonists (such as venetoclax) [4,5]. 

NF1 patients have an increased risk to develop breast cancer, gastrointestinal stromal tumor, malignant glioma, malignant peripheral nerve sheath tumor, and rhabdomyosarcoma. A breast cancer 2 (BRCA2) positive woman with NF1 who subsequently developed chronic lymphocytic leukemia is described. Chronic lymphocytic leukemia is not commonly observed in NF1 patients; the features of this woman and two other patients who had NF1 and chronic lymphocytic leukemia are reviewed [6,7].

## Case presentation

A 67-year-old woman presented for an evaluation of her skin. Her past medical history was significant for adult-onset diabetes (for which she takes metformin), BRCA2 mutation at C3362C>G, chronic lymphocytic leukemia, and NF1. She is one of 12 children; her family history is remarkable for three brothers who also have NF1 and five siblings, without NF1, who have cancer: breast cancer (two sisters, one of whom is also BRCA2 positive), chronic lymphocytic leukemia (one sister), colorectal cancer (one brother who is positive for a microsatellite instability mutation), and lung cancer (one sister).

Chronic lymphocytic leukemia (of the B-cell type) was diagnosed when she was 60 years of age. She was subsequently treated, at age 65 years, with four weekly infusions of rituximab, which--at the time of treatment--was considered to be the first-line therapy for chronic lymphocytic leukemia. She achieved a good clinical response and is currently being followed regularly by her oncologist.

**Figure 1:**
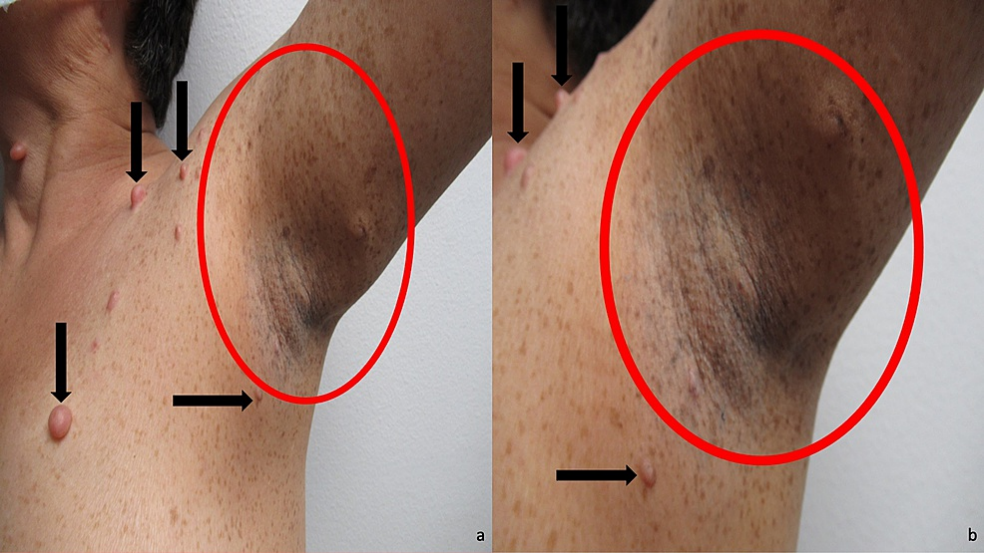
Dermatologic manifestations of neurofibromatosis type 1 Distant (a) and closer (b) views of the left neck, chest, and arm of a 67-year-old woman with neurofibromatosis type 1. Several neurofibromas (black arrows) are present.  In addition to axillary freckling (demonstrated by the two to four millimeter brown macules within the red circle), she also has many freckles that can be observed on her left chest, and left arm.

**Figure 2:**
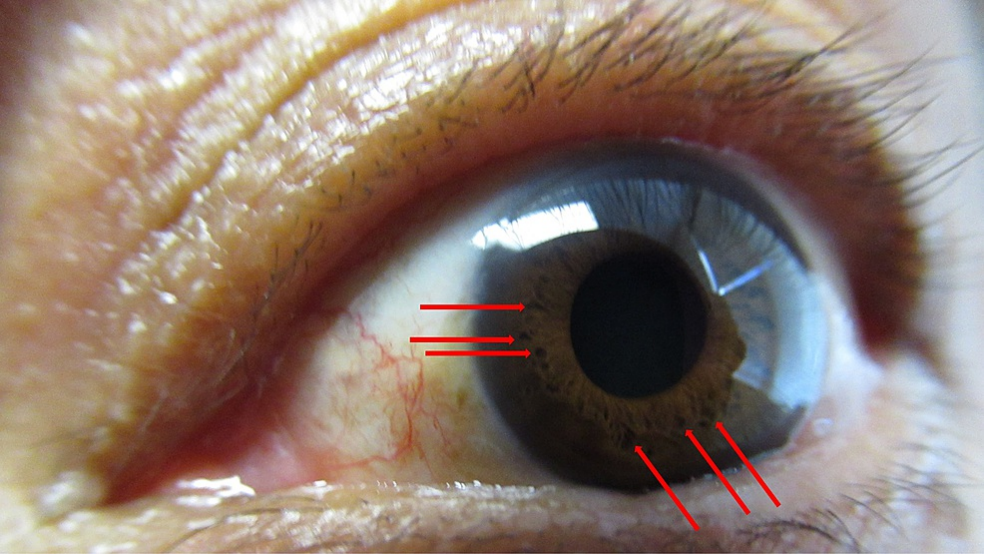
Neurofibromatosis type 1-associated Lisch nodules The iris of the left eye of a 67-year-old woman with neurofibromatosis type 1 shows many pigmented hamartomas (some of which are demonstrated by red arrows).

Cutaneous examination showed numerous soft nodules (neurofibromas) on her neck, arms, chest, and legs. Small, two to four-millimeter brown macules (freckling) were present in her left axillae and extending onto her left arm, adjacent and beneath her left breast, and on the left scapula area of her upper back (Figure 1). She had no larger brown patches (café-au-lait macules). There were several pigmented hamartomas (Lisch nodules) on the iris of both eyes (Figure 2). These clinical features, in addition to positive family history, confirmed her diagnosis of NF1. 

## Discussion

The National Institutes of Health (NIH), in 1987, proposed the criteria that are currently used to establish the diagnosis of NF1. The presence of two or more of the following criteria are necessary: (1) six or more café-au-lait macules (greater than five millimeters in diameter in prepubertal individuals and greater than 15 millimeters in postpubertal individuals); (2) two or more neurofibromas (of any type) or one plexiform neurofibroma; (3) freckling (which are small light brown colored pigmented lesions known as Crowe sign) in the axillary or inguinal regions; (4) optic pathway glioma; (5) two or more Lisch nodules (which are pigmented iris hamartomas); (6) a distinctive osseous lesion (such as sphenoid wing dysplasia--which is a defect of the skull base--or thinning of long bone cortex, with or without pseudoarthrosis); and (7) a first-degree relative (such as a parent, sibling, or offspring) with NF1 based on the above criteria. The patient in this report had four diagnostic criteria for NF1: multiple neurofibroma, axillary freckling, more than two Lisch nodules, and several siblings with NF1 [1-3].

There are several dermatologic manifestations of NF1. They include not only features that have been incorporated into the diagnostic criteria for the syndrome (such as axillary and inguinal freckling, café-au-lait macules, and neurofibromas), but also other skin conditions (such as Becker nevus, juvenile xanthogranuloma, lipoma, melanoma, nevus anemicus, nevus spilus, poliosis, and psoriasis). The patient described in this paper had both numerous neurofibromas and extensive freckling that involved not only her left axilla extending onto her left arm but also involving the scapular area of her left upper back and the skin adjacent to and beneath her left breast [1-3].

Mosaic NF1 is a localized variant of NF1 caused by somatic mosaicism, following a postzygomatic mutation, of the NF1 gene. It was previously referred to as neurofibromatosis type 5 or segmental neurofibromatosis; although both of these terms are established in earlier reports describing these individuals, the more appropriate designation of mosaic NF1 is rapidly being integrated into the current medical literature. Similar to NF1 patients, individuals with mosaic NF1 also have an increased risk to develop cancer [8,9].

Several benign and malignant tumors have been observed to occur more commonly in NF1 patients. The benign tumors include gliomas (of the optic pathway), lipomas, and neurofibromas (such as cutaneous, intraneural, and plexiform). Some of the malignant neoplasms in adults are breast cancer, gastrointestinal stromal tumor, malignant glioma (and glioma of the brainstem that behave in an aggressive manner by causing obstructive hydrocephalus), malignant peripheral nerve sheath tumor, and rhabdomyosarcoma, In children with NF1, there is an increased risk of duodenal carcinoids, pheochromocytoma, and Wilms tumor. Indeed, NF1 patients can sequentially develop multiple malignant cancers [1,3,9-11].

Children with NF1 also have an increased of developing acute leukemia and non-Hodgkin’s lymphoma. In addition, there is a 200 to 500 fold increase risk of juvenile myelomonocytic leukemia in children with NF1. Some of the NF1 children with juvenile myelomonocytic leukemia also have juvenile xanthogranuloma; several investigators consider the detection of juvenile xanthogranuloma in a child with NF1 to be a cutaneous stigmata heralding the subsequent development of juvenile myelomonocytic leukemia [1,11-13].

Chronic leukemias in adult NF1 patients are rare; indeed, there does not appear to be any genetic predisposition for either chronic myelogenous leukemia or chronic lymphocytic leukemia in NF1 patients. Chronic myelogenous leukemia, to the best of my knowledge, has only been reported in two individuals [13,14]. Similarly, including the reported woman in this paper, chronic lymphocytic leukemia has only been described in three NF1 patients: two women and one man (Table 1) [6,7].

**Table 1:** Characteristics of neurofibromatosis type 1 patients with chronic lymphocytic leukemia A, age (years, at diagnosis of CLL); AF, axillary freckling; Bld, blood; BM, bone marrow; BRCA2, breast cancer 2 positive; C, case; Calm, café-au-lait macule; CC, current case; CD, cluster of differentiation; CGS, cytogenetic studies; Chl, shlorambucil; CLL, chronic lymphocytic leukemia; CR, clinical remission; Cyc, cyclophosphamide; D, decreased; DOL, distinctive osseus lesion; FC, flow cytometry; FCR, fludarabine, cyclophosphamide, and rituximab; FHx, family history; Fi, Filipino; Fup, follow-up; G, gender; GCR, good clinical response; I, increased; In, ineffective; It, Italian; Ja, Japanese; k, thousand per microliter; LN, Lisch nodule; Lym, percent of lymphocytes (at diagnosis of CLL); M, man; Neg, negative; NF, neurofibroma; NFDC, neurofibromatosis type 1 diagnostic criteria; Nl, normal; NS, not stated; OPG, optic tract glioma; Plate, platelet count (at diagnosis of CLL); Ref, reference; Rit, rituximab; ToS, type or stage; Tx, treatment; W, woman; +, present; - absent. ^a^No chromosomal abnormalities were detected. ^b^He elected to undergo chemotherapy based on CD5/CD19 positive lymphocytosis, lymphadenopathy and thrombocytopenia. ^c^He achieved a clinical remission after treatment. ^d^Three brothers have NF1. ^e^She received four weekly infusions of Rit 41 months after her CLL diagnosis was established. ^f^She is 34 months post treatment and continues to be regularly followed by her oncologist. ^g^Her mother (who died of gastric cancer) and her son (who died of carcinoma of the small intesting) both had NF1. ^h^Studies showed a normal karyotype and no Philadelphia chromosome. ^i^She received three months of Cyc, beginning the month following CLL diagnosis; 26 months later (at age 64 years), she received four months of Chl ^j^Tx with Cyc and Chl were both ineffective. Three months after stopping Chl (at age 65 years), she developed congestive heart failure, pneumonia and an intractable increase of lymphocytes; her clinical course rapidly declined and she died five months later.

C	A Na G	NFDC Calm NF AF OPG LN DOL FHx	CLL ToS	Bld Lym	Plate	BM Lym	FC	CGS	Tx	Fup	Ref
1	48 It M	+ + + NS NS NS NS	Rai IV	I, 93	D, 82k	82	CD+: 5,19, 20,27 CD-: 7,10	Neg^a^	FCR^b^	CR^c^	[6]
2	60 Fi W	- + + - + - +^d^	B- cell	I, 85.5	Nl	87	CD+: 5 CD-: 38	BRCA2	Rit^e^	GCR^f^	CR
3	62 Ja W	+ + NS NS NS NS +^g^	B- cell	I, 97	D, 89k	85.6	CD+: 19,20, 21 CD-: 3,10	Neg^h^	Cyc^i ^ Chl^i^	In^j ^ In^j^	[7]

The NF1 patients ranged in age from 48 years to 62 years (median, 60 years) when they were diagnosed with chronic lymphocytic leukemia. The man was 48 years old. He was more than a decade younger than either of the women (who were 60 years old and 62 years old) [6,7].

The man was Italian and the women were either Filipino or Japanese [6,7]. The diagnosis of NF1 occurs equally in all ethnic groups. However, differences in penetrance and expressivity may exist; for example, pediatric brain tumors are more common in Europeans than Africans or Asians [3].

All of the patients had neurofibromas. The man and the older Japanese woman both had café-au-lait macules whereas the man and my patient, the younger Filipino woman, both had axillary freckling. Both my patient and the older Japanese woman had a family history of NF1--either three brothers or a mother and son. The only other diagnostic feature of NF1 that my patient, the younger woman, had was Lisch nodules [6,7].

My patient and the other woman both had B-cell chronic lymphocytic leukemia. All of the patients had elevated percentage of lymphocytes in the peripheral blood and bone marrow. The man and the older Japanese woman also had thrombocytopenia [6,7].

Flow cytometry was performed. The lymphocytes of both the man and my patient, the younger woman, expressed CD5. The lymphocytes of both the man and the older Japanese woman expressed cluster of differentiation 19 (CD19) and CD20. In addition expression of cluster of differentiation 21 (CD21) and cluster of differentiation 27 (CD27) were observed on the lymphocytes of the older Japanese woman and the man, respectively [6,7].

Cytogenetic studies were also performed. They were negative for the man (demonstrating no chromosome abnormalities) and the older Japanese woman (showing a normal karyotype and no Philadelphia chromosome). In contrast, my patient, the younger woman, was found to be BRCA2 positive [6,7].

The severity of the leukemia was advanced for the man (Rai stage IV) and the older Japanese woman. Both were treated immediately after the diagnosis of chronic lymphocytic leukemia was established. In contrast, my patient (the younger Filipino woman) was monitored clinically for nearly three and a half years before receiving treatment for her leukemia [6,7]. 

All of the patients were eventually treated with antineoplastic agents; the diagnosis of NF1 did not influence the chronic lymphocytic leukemia management. The man achieved clinical remission after receiving combination therapy consisting of fludarabine, cyclophosphamide, and rituximab. My patient, the younger woman, had a good clinical response after four weekly infusions of rituximab; she remains on periodic follow-up observation 34 months after treatment without significant progression of her leukemia. The older Japanese woman died of progressive neoplastic disease; after sequentially failing cyclophosphamide and chlorambucil, she developed congestive heart failure, pneumonia, and intractable lymphocytosis [6,7].

The currently reported patient is BRCA2 positive. Breast cancer 1 (BRCA1) and BRCA2 mutations increase the individual’s lifetime risk of breast cancer (in both women and men) and ovarian cancer--in addition to the fallopian tube and primary peritoneal cancers. An increased risk to develop melanoma, pancreatic cancer, and prostate cancer has also been shown in BRCA2 positive individuals [15].

Similar to the NF1 gene, BRCA1 is located on chromosome 17 (17q21). In contrast, BRCA2 is located on chromosome 13 (13q12). However, positive mutation for BRCA1 or BRCA2 in NF1 patients is not commonly described [15-18].

My patient, the BRCA2 positive woman in this report, did not have breast or ovarian cancer. Yet, BRCA1 positivity was demonstrated by researchers in a woman with NF1 who also had breast cancer. Also, in a study of 78 NF1 women with breast cancer, the investigators evaluated BRCA1 and BRCA2 mutations in 18 of the patients and were only able to demonstrate a BRCA2 mutation in one individual [16-18].

However, my patient (the BRCA2 positive NF1 patient described in this paper) had chronic lymphocytic leukemia. Mutations of either BRCA1 or BRCA2 has also been demonstrated to increase the risk for acute leukemias (such as acute lymphocytic leukemia and acute myelogenous leukemia), chronic leukemias (such as chronic lymphocytic leukemia and prolymphocytic leukemia), and mantle cell lymphoma. Investigators have previously observed a 67-year-old BRCA2 positive woman who developed--during a period of 30 months--not only chronic lymphocytic leukemia, but also three other primary malignancies: breast ductal carcinoma, endocervical adenocarcinoma, and ovarian serous papillary carcinoma [19,20].

## Conclusions

NF1 is a genodermatosis with malignant potential; adult patients with the syndrome have an increased lifetime risk to develop malignancies such as breast cancer, gastrointestinal stromal tumor, malignant glioma, malignant peripheral nerve sheath tumor, and rhabdomyosarcoma. A BRCA2 positive woman with NF1 and chronic lymphocytic leukemia are described; including my younger female Filipino patient in this paper, chronic lymphocytic leukemia has only been reported in two women and one man with NF1. The man (who achieved a clinical remission) and the older Japanese woman (who died secondary to complications associated with therapy-refractory progression of her leukemia) presented with advanced chronic lymphocytic leukemia and required antineoplastic treatment at diagnosis. My female patient’s chronic lymphocytic leukemia had a good clinical response to treatment 41 months after diagnosis which was sustained without significant disease progression for 34 months. An increased lifetime risk of developing cancer--particularly breast and ovarian carcinoma--are associated with BRCA1 and BRCA2 mutation; in addition, patients with mutations of either BRCA1 or BRCA2 have an increased risk of chronic lymphocytic leukemia. Also, albeit uncommon, women with NF1 who develop breast cancer may have either BRCA1 or BRCA2 mutation. In conclusion, it remains to be established whether chronic lymphocytic leukemia in NF1 patients is related to the increased risk of individuals with the genodermatosis to develop malignancy or whether the observation of this leukemia in three individuals with NF1 is merely a coincidence; however, it is possible that the occurrence of chronic lymphocytic leukemia in the woman with NF1 who has been reported in this paper may, in part, be related to her BRCA2 positivity.

## References

[1] Gutmann DH, Ferner RE, Listernick RH, Korf BR, Wolters PL, Johnson KJ (2017). Neurofibromatosis type 1. Nat Rev Dis Primers.

[2] Miraglia E, Moliterni E, Iacovino C, Roberti V, Laghi A, Moramarco A, Giustini S (2020). Cutaneous manifestations in neurofibromatosis type 1. Clin Ter.

[3] Wilson BN, John AM, Handler MZ, Schwartz RA (2020). Neurofibromatosis type 1: new developments in genetics and treatment. J Am Acad Dermatol.

[4] Chiorazzi N, Chen SS, Rai KR (2021). Chronic lymphocytic leukemia. Cold Spring Harb Perspect Med.

[5] Burger JA (2020). Treatment of chronic lymphocytic leukemia. N Engl J Med.

[6] Miraglia E, Iacovino C, Cantisani C, Calvieri S, Giustini S (2016). An unusual manifestation in a patient with neurofibromatosis type 1. G Ital Dermatol Venereol.

[7] Sanada M, Takai K, Shibuya H, Okazaki E (1991). Chronic lymphocytic leukemia associated with von Recklinghausen neurofibromatosis. Int J Hematol.

[8] García-Romero MT, Parkin P, Lara-Corrales I (2016). Mosaic neurofibromatosis type 1: a systematic review. Pediatr Dermatol.

[9] Cohen PR (2016). Segmental neurofibromatosis and cancer: report of triple malignancy in a woman with mosaic neurofibromatosis 1 and review of neoplasms in segmental neurofibromatosis. Dermatol Online J.

[10] Przybylik-Mazurek E, Palen J, Pasternak-Pietrzak K, Sowa-Staszczak A, Brzozowska-Czarnek A, Hubalewska-Dydejczyk A (2018). Coexistence of neurofibromatosis type 1 with multiple malignant neoplasia. Neuro Endocrinol Lett.

[11] Lin AL, Gutmann DH (2013). Advances in the treatment of neurofibromatosis-associated tumours. Nat Rev Clin Oncol.

[12] Paulus S, Koronowska S, Fölster-Holst R (2017). Association between juvenile myelomonocytic leukemia, juvenile xanthogranulomas and neudrofibromatosis type 1: case report and review of the literature. Pediatr Dermatol.

[13] Olayemi EE, Benneh AA, Acquah ME, Mensah PK (2011). Chronic myeloid leukemia in an adult ghanaian with sporadic neurofibromatosis 1. Indian J Dermatol.

[14] Gulhane SR, Kotwal MN (2015). Chronic myeloid leukemia arising in a patient of neurofibromatosis type 1. Indian J Dermatol.

[15] Sabiani L, Barrou J, Mathis J, Eisinger F, Bannier M, Lambaudie E, Houvenaeghel G (2020). How to manage BRCA mutation carriers?. Horm Mol Biol Clin Investig.

[16] Seo YN, Park YM (2015). Association between neurofibromatosis type 1 and breast cancer: a report of two cases with a review of the literature. Case Rep Med.

[17] Jeon YW, Kim RM, Lim ST, Choi HJ, Suh YJ (2015). Early-onset breast cancer in a family with neurofibromatosis type 1 associated with a germline nutation in BRCA 1. J Breast Cancer.

[18] Frayling IM, Mautner VF, van Minkelen R (2019). Breast cancer risk in neurofibromatosis type 1 is a function of the type of <i>NF1</i> gene mutation: a new genotype-phenotype correlation. J Med Genet.

[19] Friedenson B (2007). The BRCA1/2 pathway prevents hematologic cancers in addition to breast and ovarian cancers. BMC Cancer.

[20] Fruscalzo A, Damante G, Calcagno A, Di Loreto C, Marchesoni D (2006). Four primary malignancies successively occurred in a BRCA2 mutation carrier: a case report. Cancer Invest.

